# Patterns of Cell Activity in the Subthalamic Region Associated with the Neuroprotective Action of Near-Infrared Light Treatment in MPTP-Treated Mice

**DOI:** 10.1155/2012/296875

**Published:** 2012-05-14

**Authors:** Victoria E. Shaw, Cassandra Peoples, Sharon Spana, Keyoumars Ashkan, Alim-Louis Benabid, Jonathan Stone, Gary E. Baker, John Mitrofanis

**Affiliations:** ^1^Discipline of Anatomy and Histology, The University of Sydney, Sydney, NSW 2006, Australia; ^2^Deptartment of Neurosurgery, King's College Hospital, London SE59RS, UK; ^3^Clinatec LETI-DTBS, CEA, 38054 Grenoble, France; ^4^Discipline of Physiology, The University of Sydney, Sydney, NSW 2006, Australia; ^5^Deptartment of Optometry and Visual Science, City University London, London EC1VOHB, UK

## Abstract

We have shown previously that near-infrared light (NIr) treatment or photobiomodulation neuroprotects dopaminergic cells in substantia nigra pars compacta (SNc) from degeneration induced by 1-methyl-4-phenyl-1,2,3,6-tetrahydropyridine (MPTP) in mice. The present study explores whether NIr treatment changes the patterns of Fos expression in the subthalamic region, namely, the subthalamic nucleus (STN) and zona incerta (ZI); both cell groups have abnormally overactive cells in parkinsonian cases. BALB/c mice were treated with MPTP (100–250 mg/kg) or saline either over 30 hours followed by either a two-hour or six-day survival period (acute model) or over five weeks followed by a three-week survival period (chronic model). NIr and MPTP were applied simultaneously. Brains were processed for Fos immunochemistry, and cell number was estimated using stereology. Our major finding was that NIr treatment reduced (30–45%) the increase in Fos^+^ cell number evident in the STN and ZI after MPTP insult. This reduction was concurrent with the neuroprotection of dopaminergic SNc cells shown previously and was evident in both MPTP models (except for the 2 hours survival period which showed no changes in cell number). In summary, our results indicated that NIr had long lasting effects on the activity of cells located deep in the brain and had repaired partially the abnormal activity generated by the parkinsonian toxin.

## 1. Introduction

Exposure to near-infrared light treatment (NIr), also referred to as photobiomodulation, has been shown to protect dopaminergic cells from degeneration induced by 1-methyl-4-phenyl-1,2,3,6-tetrahydropyridine (MPTP) in both *in vitro* [2, 3] and *in vivo* (within substantia nigra pars compacta; SNc) [4, 5] studies. This neuroprotection is presumably due to the NIr limiting the mitochondrial dysfunction and subsequent oxidative stress and free-radical production caused by the MPTP, which induces Parkinson-like pathology [4, 6]. NIr has been shown to improve mitochondrial function and ATP (adenosine-5′-triphosphate) production by increasing the electron transfer in the respiratory chain and activation of photoacceptors, such as cytochrome oxidase [7, 8]. Although these results are from *in vitro* studies [2, 3] and from an animal model of the disease [4, 5], the outcome is potentially exciting; that a noninvasive procedure offers neuroprotection in Parkinson's disease, a feature that most current forms of treatment, including dopamine-replacement drug therapy, does not do [9].

In this study, we have sought to extend our earlier anatomical findings [4, 5] by examining patterns of cell activation associated with the neuroprotective action of NIr in parkinsonian cases. To this end, we examined cell activity in the subthalamic region, namely, the subthalamic nucleus (STN) and the zona incerta (ZI). We chose this region for two main reasons (i) STN and ZI cells have abnormal overactivity in parkinsonian cases [10–13] and (ii) both cell groups have become a popular targets for surgical intervention, particularly with deep brain stimulation [14–16]. It is well accepted that an increase in Fos expression, after activation of the cFos gene, reflects an increase in cell activity. This method has been used to study global cell activity patterns in many brain regions after various forms of stimulation and disease states, including parkinsonism [17, 18]. In essence, we tested whether NIr treatment was able to influence the activity of cells located deep in the brain and reverse the abnormal activity induced by the parkinsonian insult.

## 2. Materials and Methods

### 2.1. Subjects

Male albino BALB/c mice (~20 g; ~8-week old; *n* = 96) were housed on a 12 hours light/dark cycle with unlimited access to food and water. All experiments were approved by the Animal Ethics Committee of The University of Sydney.

### 2.2. Experimental Design

An acute [4, 19] and a chronic [5] MPTP model were used in this study. There were four experimental groups in each model, where mice received intraperitoneal injections of either MPTP or saline, combined with simultaneous NIr treatments or not. The different groups were (1) *saline* (*n* = 24): saline injections with no NIr, (2) *saline-NIr* (*n* = 24): saline injections with NIr, (3) *MPTP* (*n* = 24): MPTP injections with no NIr, and (4) *MPTP-NIr* (*n* = 24): MPTP injections with NIr.

For the acute model, four (25 mg/kg injections; total of 100 mg/kg per mouse) MPTP or saline injections were made over a 30-hour period. After the last injection, mice were allowed to survive for either two hours (*n* = 32) or six days (*n* = 32). For the chronic model (*n* = 32), mice had ten injections of MPTP (20 mg/kg per injection; total of 200 mg/kg per mouse) or saline combined with probenecid (250 mg/kg; decreases renal excretion of MPTP and hence maintains the effects of toxin during injection intervals) approximately three and a half days apart, over a five-week period. After the last injection, mice were allowed to survive for three weeks. For both models, the dose regimes and survival periods were the same as those used by previous studies [4, 5, 19]. The survival periods were selected as to determine whether there were any immediate or longer lasting changes in Fos expression after MPTP (or NIr) treatment.

For the NIr treatment, mice in the MPTP-NIr and saline-NIr groups of each model (acute and chronic) were treated with 670 nm light from a light-emitting device (LED; Quantum Devices WARP 10) as described previously [4, 5]. Briefly, mice had NIr treatment (one cycle of 90 seconds; estimated at 0.5 Joule/cm^2^ to the brain [4]) for ~15 minutes after each MPTP or saline injection. Hence, for each MPTP insult there would be almost immediate potential therapeutic application. For both models, these NIr treatment regimes were similar to that used by previous studies, in particular, those reporting changes after transcranial irradiation [4, 5, 20, 21]. For each exposure to NIr, the mouse was restrained gently by hand, and the LED was held 1-2 cm above the head [4, 5]. The mice tended to relax during exposure, and reliable delivery of the radiation was achieved readily. The LED generated very little heat and it did not cause the mice any visible discomfort. For the saline and MPTP groups, mice were held under the LED as described above for 90 seconds, but the device was not turned on [4, 5].

Our experimental paradigm, of essentially simultaneous administration of parkinsonian insult and therapeutic application, was similar to that of many previous studies on animal models of Parkinsons disease [4, 5, 19, 22]. This paradigm is unlike the clinical reality where considerable dopaminergic cell loss occurs prior to therapeutic intervention. However, in our experimental study—as with the abovementioned previous ones—we hoped to explore the maximum effect of NIr treatment on the number of dopaminergic cells and hence determine more systematically its effects on functional activity (i.e., Fos expression).

### 2.3. Immunocytochemistry

Following the survival periods, mice were anaesthetised with an intraperitoneal injection of sodium pentobarbital (60 mg/mL). They were then perfused transcardially with 0.1 M phosphate-buffered saline (PBS; pH 7.4), followed by 4% buffered formaldehyde. The brains were removed and postfixed overnight in the same solution. Next, brains were placed in PBS with the addition of 30% sucrose until the block sank. The forebrain was then sectioned coronally and serially using a freezing microtome. All sections were collected in PBS and then immersed in a solution of 1% Triton (Sigma) and 10% normal goat serum (Sigma) at room temperature for 30 minutes each. Sections were then incubated in anti-cFos (SantaCruz; 1 : 4000) for 48 hours (at 4°C), followed by biotinylated anti-rabbit IgG (Bioscientific; 1 : 200) for three hours (at room temperature), and then streptavidin-peroxidase complex (Bioscientific; 1 : 200) for two hours (at room temperature). To visualise the bound antibody, sections were reacted in nickel-Tris-buffered saline (pH 7.4)-3,3′-diaminobenzidine tetrahydrochloride (Sigma) solution. In between incubations, sections were washed in three changes of PBS. Sections were mounted onto gelatinised slides, air dried overnight, dehydrated in ascending alcohols, cleared in Histoclear, and coverslipped using DPX. Most of our immunostained sections were counterstained lightly with neutral red as well. For controls, sections were processed as described above, except that there was no primary antibody used. These control sections were immunonegative.

### 2.4. Analysis

Following the procedures outlined by previous studies [4, 5, 19], the number of Fos^+^ cells within the STN and ZI were estimated using the optical fractionator method (StereoInvestigator, MBF Science). Briefly, systematic random sampling of sites within defined boundaries of the STN and ZI was undertaken. All cells that came into focus within the frame were counted. Figures 4(e) and 5(e) show schematic diagrams of the mouse brain, and the shaded areas indicate the general regions that were analysed. The distribution maps of Fos^+^ cells (Figures 4(a)–4(d), 5(a)–5(d)) were constructed using the StereoInvestigator programme also. For comparisons between groups within each model (six-day survival and two-hour survival acute model and chronic model), a one-way ANOVA test (*F* and *P* values) was performed, in conjunction with a Tukey-Kramer multiple comparison test (*q* and *P* values) (using GraphPad Prism programme). Schematic diagrams and digital images were constructed using Adobe Photoshop and Microsoft PowerPoint programmes.

## 3. Results

In what follows, the morphology, number, and distribution of Fos^+^ cells in the STN and ZI will be considered separately. 

### 3.1. Morphology

**Figure 1:**
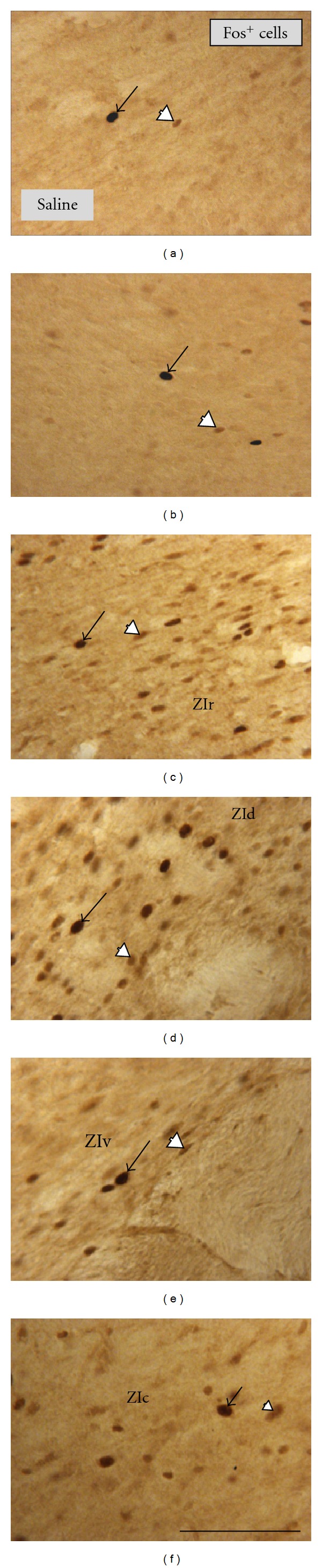
Fos^+^ cells in the STN (central region) and in the different sectors of the ZI, namely, ZIr (a), ZId (b), ZIv (c), and ZIc (d). Fos immunoreactivity was apparent in cell nuclei; cells were either strongly (black arrows) or weakly immunostained (white arrowheads). All cases are from the saline group; patterns of immunostaining were similar in the other groups of both acute and chronic models. All figures are of coronal sections; dorsal to top and lateral to right. Scale bar  =  100 *μ*m.

In both the STN and ZI, Fos immunoreactivity was limited to the nuclei of cells (Figure 1). The intensity of immunoreactivity was not consistent across both cell groups; cells were immunostained either strongly (arrows, Figure 1) or weakly (arrowheads, Figure 1). These patterns were evident across the STN (Figures 1(a) and 1(b)) and in each of the different sectors of the ZI, namely, rostral (Figure 1(c)), dorsal (Figure 1(d)), ventral (Figure 1(e)), and caudal (Figure 1(f)) and were similar in all the experimental groups of both acute and chronic MPTP models.

### 3.2. Number

In this study, we counted the number of Fos^+^ cells in the STN and ZI that were immunostained strongly (see above), presumably because they had undergone the most activation [17, 23]. The number of cells in the STN and ZI in the acute and chronic MPTP models will be considered separately below. 

#### 3.2.1. Subthalamic Nucleus (STN)

**Figure 2:**
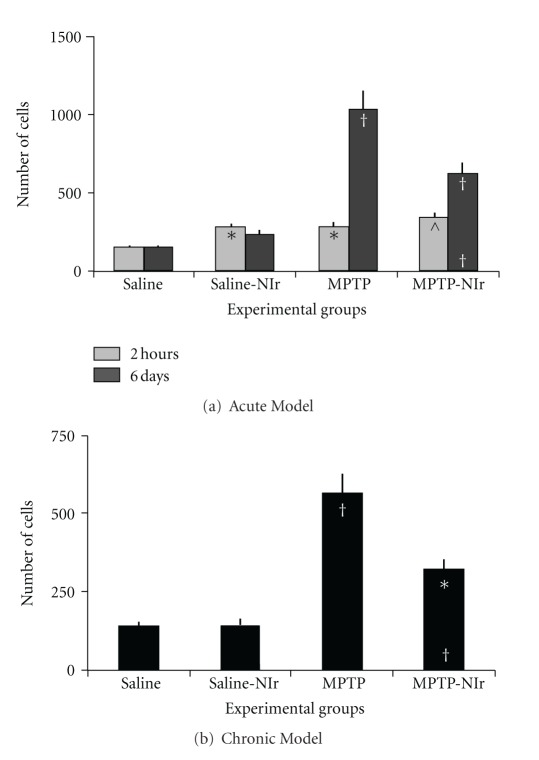
Graphs showing number of Fos^+^ cells in the STN of the four groups in the acute (a) and chronic (b) MPTP mouse models. Columns show the mean ± standard error of the total number of cells in each group. In (a) and (b), † represents *P* < 0.001 and ^∧^ represent *P* < 0.01 significant difference in cell number between different groups (using Tukey-Kramer multiple comparison test). The symbols at the top of each column represents differences with the saline group, while the symbols at the bottom of MPTP-NIr group column represent differences with the MPTP group.

The graph in Figure 2(b) shows the estimated number of Fos^+^ cells in the STN of the four experimental groups in the acute MPTP model. In the two-hour survival period, there were 50–60% more Fos^+^ cells in the saline-NIr, MPTP, and MPTP-NIr groups than in the saline group. Overall, using an ANOVA test, these differences were significant (*F* = 6.2; *P* < 0.001). Using the Tukey-Kramer test, significant differences in total number were found between the saline group and the saline-NIr (*q* = 4.0; *P* < 0.05), MPTP (*q* = 4.1; *P* < 0.05), and MPTP-NIr (*q* = 5.9; *P* < 0.01) groups. There were no significant differences between the saline-NIr group and the MPTP (*q* = 0.1; *P* > 0.05) or MPTP-NIr (*q* = 1.9; *P* > 0.05) groups, nor between the MPTP and MPTP-NIr groups (*q* = 1.8; *P* > 0.05). In the six-day survival period, the MPTP group had more Fos^+^ cells than the MPTP-NIr (40%), saline-NIr (80%), and saline (85%) groups. Of particular relevance was that the MPTP-NIr group had fewer Fos^+^ cells than the MPTP group, indicating that NIr treatment had reduced the Fos expression induced by the MPTP insult. Further, the MPTP-NIr group had more Fos^+^ cells than the saline groups (65–75%), indicating that although there was a reduction in number from the MPTP insult, it did not quite reach control levels. Overall, these differences between the groups were significant using an ANOVA test (*F* = 65.7; *P* < 0.0001). Using the Tukey-Kramer test, significant differences in total number were found between the MPTP group and saline (*q* = 17.6; *P* < 0.001), saline-NIr (*q* = 16.0; *P* < 0.001), and, notably, MPTP-NIr (*q* = 8.1; *P* < 0.001) groups. There were also significant differences between the MPTP-NIr group and saline (*q* = 9.5; *P* < 0.001) and saline-NIr (*q* = 7.9; *P* < 0.001) groups. The differences in total number between the saline and saline-NIr groups were not significant (*q* = 1.6; *P* > 0.05).

The graph in Figure 2(b) shows the estimated number of Fos^+^ cells in the STN of the four groups in the chronic MPTP model. The patterns of Fos^+^ cell number shown for this model were similar to those described above for the six-day survival acute model. The MPTP group had more Fos^+^ cells than the MPTP-NIr (45%), saline-NIr (75%), and saline (75%) groups. The MPTP-NIr group had fewer Fos^+^ cells than the MPTP group but many more cells than in the saline groups (55%). Overall, these differences between the groups were significant using an ANOVA test (*F* = 34.2; *P* < 0.0001). Using the Tukey-Kramer test, significant differences in total number were found between the MPTP group and saline (*q* = 12.4; *P* < 0.001), saline-NIr (*q* = 12.3; *P* < 0.001), and MPTP-NIr (*q* = 7.1; *P* < 0.001) groups. There were also significant differences between the MPTP-NIr group and the saline (*q* = 5.4; *P* < 0.01) and saline-NIr (*q* = 5.2; *P* < 0.01) groups. The differences in total number between the saline and saline-NIr groups were not significant (*q* = 0.2; *P* > 0.05).

It should be noted that the number of tyrosine hydroxylase (TH)^+^ cells in the substantia nigra pars compacta (SNc), from the same brains from as those used here for Fos immunocytochemistry, have been analysed also, and full details of the results have been published [4, 5]. Briefly, these studies showed substantial TH^+^ cell loss in the SNc in both acute (~60%, six-day survival; no change in TH^+^ cell number in two-hour survival period) and chronic (~45%) MPTP models. In addition, there was also fewer TH^+^ terminals in the striatum, the major termination zone of the SNc axons, of the MPTP groups compared to the others. Finally, the MPTP-NIr groups of both models had more TH^+^ cells than in the MPTP group (30–35%) but not quite as many as in the saline control groups (25–30%).

#### 3.2.2. Zona Incerta (ZI)

**Figure 3:**
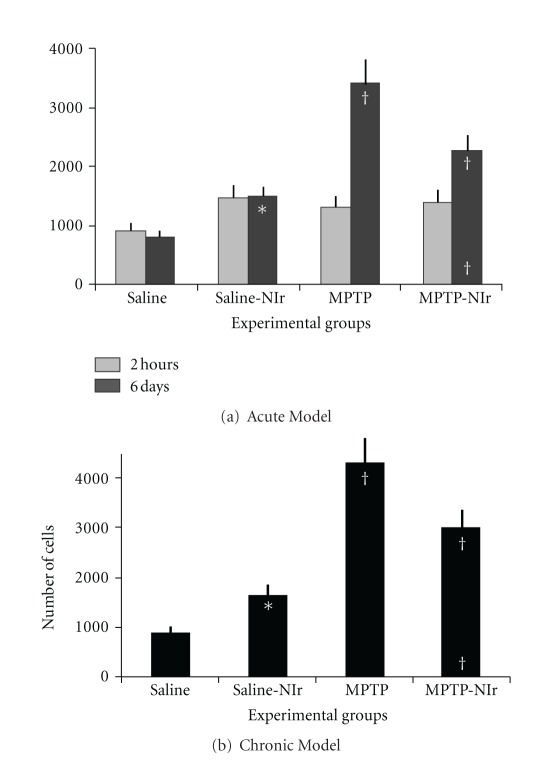
Graphs showing number of Fos^+^ cells in the ZI of the four groups in the acute (a) and chronic (b) MPTP mouse models. Columns show the mean ± standard error of the total number of cells in each group. In (a) and (b), † represents *P* < 0.001 and ^∧^ represent *P* < 0.01 significant difference in cell number between different groups (using Tukey-Kramer multiple comparison test). The symbols at the top of each column represents differences with the saline group, while the symbols at the bottom of MPTP-NIr group column represent differences with the MPTP group.

The graph in Figure 3(a) shows the estimated number of Fos^+^ cells in the ZI of the four experimental groups in the acute MPTP model. In the two-hour survival period, there were no significant differences between the number of Fos^+^ cells in the different groups using an ANOVA test (*F* = 2.4; *P* > 0.05). Using the Tukey-Kramer test, no significant differences in total number were found between the saline group and saline-NIr (*q* = 3.4; *P* > 0.05), MPTP (*q* = 2.6; *P* > 0.05), and MPTP-NIr (*q* = 3.0; *P* > 0.05) groups, between the saline-NIr group and MPTP (*q* = 0.9; *P* > 0.05), and MPTP-NIr (*q* = 0.5; *P* > 0.05) groups, nor between the MPTP and MPTP-NIr groups (*q* = 0.4; *P* > 0.05). In the six-day survival period, the MPTP group had many more Fos^+^ cells than the MPTP-NIr (35%), saline-NIr (55%), and saline (75%) groups. Of particular relevance was that the MPTP-NIr group had fewer Fos^+^ cells than the MPTP group but more than the saline (65%) and saline-NIr (35%) groups. Overall, these differences between the groups were significant using an ANOVA test (*F* = 43.3; *P* < 0.0001). Using the Tukey-Kramer test, significant differences in total number were found between the MPTP group and saline (*q* = 15.4; *P* < 0.001), saline-NIr (*q* = 11.2; *P* < 0.001), and, notably, MPTP-NIr (*q* = 6.8; *P* < 0.001) groups. There were also significant differences between the saline group and the saline-NIr (*q* = 4.2; *P* < 0.05) and MPTP-NIr (*q* = 8.6; *P* < 0.001) groups and between the MPTP-NIr and Saline-NIr groups (*q* = 4.5; *P* < 0.05).

The graph in Figure 3(b) shows the estimated number of Fos^+^ cells in the ZI of the four groups in the chronic MPTP model. The patterns of Fos^+^ cell number shown for this model were similar to those described above for the six-day survival acute model. The MPTP group had more Fos^+^ cells than the MPTP-NIr (30%), saline-NIr (60%), and saline (80%) groups. The MPTP-NIr group had fewer Fos^+^ cells than the MPTP group but many more cells than the saline (70%) and saline-NIr (45%) groups. Overall, these differences between the groups were significant using an ANOVA test (*F* = 66.8; *P* < 0.0001). Using the Tukey-Kramer test, significant differences in total number were found between the MPTP group and saline (*q* = 18.5; *P* < 0.001), saline-NIr (*q* = 14.4; *P* < 0.001), and MPTP-NIr (*q* = 7.0; *P* < 0.001) groups. There were also significant differences between the MPTP-NIr group and the saline (*q* = 11.5; *P* < 0.001) and saline-NIr (*q* = 7.4; *P* < 0.001) groups and between the saline and saline-NIr groups (*q* = 4.1; *P* < 0.05).

In summary, MPTP insult generated a substantial increase in the number of Fos^+^ cells in both the STN and ZI, and NIr treatment reduced this increase significantly. These changes were concurrent with changes in TH^+^ cell number in the SNc in the acute model with six-day survival period and chronic model [4, 5]. Unlike in the STN, there was a significant increase in the number of Fos^+^ cells in the ZI after NIr treatment alone.

## 4. Distribution

### 4.1. Subthalamic Nucleus (STN)

**Figure 4:**
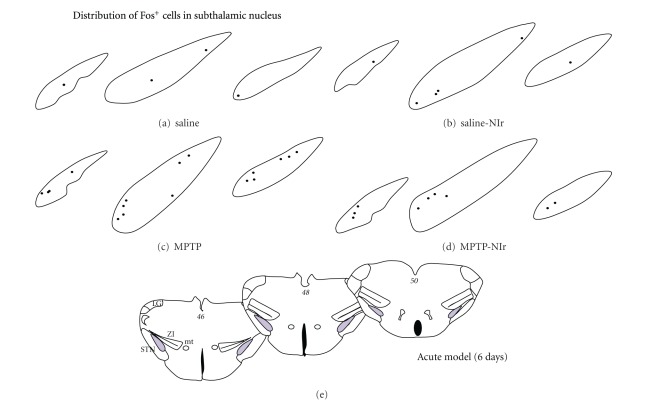
Schematic diagrams of the distribution of Fos^+^ cells in the different sectors of the STN in the saline (a), saline-NIr (b), MPTP (c) and MPTP-NIr groups. These maps are from the acute model with six-day survival period (similar patterns were evident in the chronic model; in the acute model with two-hour survival period, the distribution of cells was similar to the saline cases shown here). Each black circle represents one cell. These maps were taken from coronal sections similar to the plates of the mouse atlas (numbers in italics [1]) shown in (e).

Figure 4 shows schematic diagrams of the distribution of Fos^+^ cells in the STN in the acute MPTP model with six-day survival period (distributions were similar in the chronic model; in the two-hour survival period, the distributions in all the groups were similar to the saline group). In all groups, Fos^+^ cells tended to be scattered across the nucleus, with no clear zone of concentration. There were more Fos^+^ cells evident in the MPTP-NIr and, in particular, the MPTP group than in the saline controls (see above).

### 4.2. Zona Incerta (ZI)

**Figure 5:**
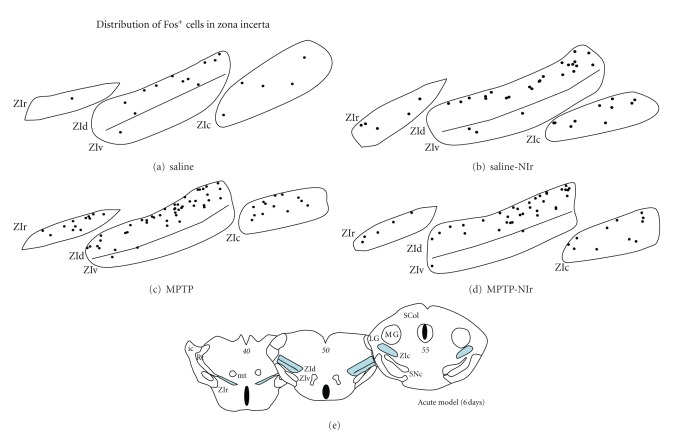
Schematic diagrams of the distribution of Fos^+^ cells in the different sectors of the STN in the saline (a), saline-NIr (b), MPTP (c) and MPTP-NIr groups. These maps are from the acute model with six-day survival period (similar patterns were evident in the chronic model; in the acute model with two-hour survival period, the distribution of cells was similar to the saline cases shown here). Each black circle represents one cell. These maps were taken from coronal sections similar to the plates of the mouse atlas (numbers in italics [1]) shown in (e).

Figure 5 shows schematic diagrams of the distribution of Fos^+^ cells in the ZI and its sectors (rostral, dorsal, ventral, and caudal) of the different groups in the acute MPTP model with six-day survival period (as with STN, distributions were similar in other models). In the saline group, Fos^+^ cells were scattered sparsely across each incertal sectors but with a tendency to concentrate in the dorsal sector (Figure 5(a)). In the saline-NIr (Figure 5(b)), MPTP (Figure 5(c)), and MPTP-NIr (Figure 5(d)) groups, Fos^+^ cells were also found somewhat scattered, albeit in higher numbers, across each of the sectors. In these groups, the same concentration in the dorsal sector was evident.

In summary, the Fos^+^ cells in the STN showed no clear pattern of topography across the nucleus, while in the ZI, most cells were located in the dorsal sector.

## 5. Discussion

We had three main findings. First, unlike the STN, there were many ZI cells that expressed the Fos protein after NIr treatment. Second, in both the STN and ZI, MPTP insult generated a substantial increase in the number of Fos^+^ cells. Finally, and most notably, NIr treatment reduced this increase in STN and ZI Fos^+^ cell number after MPTP insult; this reduction was concurrent with the neuroprotection of TH^+^ cells in the SNc. These differences in Fos^+^ cell number were evident in the longer-term survival periods (six days to three weeks) of acute and chronic models, indicating that NIr (and MPTP) treatment had long lasting effects on neuronal function. Each of these issues will be explored below. First, a comparison with previous studies will be considered.

### 5.1. Comparison with Previous Studies

To the best of our knowledge, there has been no other study that has examined Fos expression in the STN and ZI, nor indeed the brain, after NIr treatment. Several previous studies, using different methods and animal models of Parkinson's disease, have reported on the patterns of cell activity in the STN and ZI. These studies have used either metabolic markers and/or electrophysiological methods in 6-OHDA-lesioned rats [10, 11, 13] or MPTP-treated monkeys [12]. We confirm these previous reports of an increase in cell activity in STN and ZI by using Fos immunocytochemistry in MPTP-treated mice. It should be noted that somewhat different results have been reported in 6-OHDA-lesioned rats using Fos immunocytochemistry in these nuclei. In these studies, very few Fos^+^ cells were seen in the STN [24] or ZI [25] after 6-OHDA lesion. The reason for this difference in our studies is not entirely clear, but it may be related to the different animals used (6-OHDA versus MPTP)—and there have been many reports of dissimilar results using the different models [26]—together with the location of the 6-OHDA injection site. In the previous studies, 6-OHDA was injected into the medial forebrain bundle which lies immediately adjacent to the STN and ZI. There could have been some spread of the 6-OHDA toxin from the injection site into the adjacent STN and ZI, and this may have influenced the expression of Fos in these cases. Indeed, in cases where 6ODHA was injected directly into the SNc, a region well away from the STN and ZI, an increase in cellular STN and ZI activity was apparent [10–13]. Perhaps 6-OHDA injections into the SNc or striatum, regions well away from the STN and ZI, may generate results similar to ours seen here in MPTP-treated mice.

### 5.2. NIr Treatment Induced Fos Expression in Zona Incerta

One of our most striking, and least expected, findings was of an increase in Fos expression within the ZI but not the STN after the application of NIr treatment alone. Two mechanisms for this increase in expression can be suggested. First, NIr could have activated an excitatory afferent pathway, one that terminates in the ZI and not the STN, triggering Fos expression. Such a pathway was likely to originate from the superior colliculus, which has a very heavy projection to the ZI (in particular to its dorsal sector), but not the STN [27–29]. The superior colliculus receives a large and direct input from the retina, whose cells may have been activated directly by NIr. Further, the retina has a direct, albeit very small, input to the ZI but not to the STN [27, 29]. This small input, through an extensive network of inter-ZI connections [29], may have contributed to the Fos expression in the ZI as well. Second, NIr could have activated directly the mitochondria within the ZI cells themselves, inducing Fos expression. The ZI cells may have been more susceptible to photoacceptor (cytochrome oxidase) activation than the cells in the STN. It remains to be determined which other cell groups in the central nervous system are activated by NIr treatment alone, and how they may relate to the activated ZI cells described here.

### 5.3. Fos Expression after MPTP Treatment

We report that MPTP insult induced Fos expression in the STN and ZI. In fact, the MPTP group had more Fos^+^ cells in both nuclei than any one of the other groups. This activation was likely to manifest after the loss of SNc dopaminergic cells caused by the MPTP [10–13]. For the ZI, it receives a very weak direct projection from the SNc, hence the activation was likely to occur indirectly via, for example, the PpT, which is overactive in parkinsonian cases and has heavy projections to the ZI [29]. For the STN, it receives a more substantial SNc projection, together with heavy projections from the PpT [27, 28], and hence these projections may have both contributed to the Fos expression in this nucleus.

Hence, there appear to be two very different triggers or mechanisms that generated the Fos expression. In the MPTP group, Fos expression in both the STN and ZI was likely to be induced after a loss of dopaminergic cells from the SNc (and overactive PpT projections), while in the saline-NIr group, a group that had no loss of SNc cells, Fos expression in the ZI may have been induced by pathways stimulated by retinal afferents (via the superior colliculus) after the NIr treatment.

### 5.4. NIr Treatment Reduced Abnormal Fos Expression Induced by MPTP Insult: A Long-Lasting Effect

Our results showed that neuroprotection or saving of dopaminergic cells in the SNc from MPTP toxicity by NIr treatment [4, 5] resulted in a reduction of the abnormal overactivity (increase in Fos expression) in the STN and ZI. There were fewer Fos^+^ cells in the MPTP-NIr compared to the MPTP group. The number of cells in the MPTP-NIr group was, however, still higher than in the saline-NIr (30–45%) and saline (60%) control groups, indicating that the NIr treatment did not reduce entirely the abnormal activity generated by the MPTP toxin; if it did, then the MPTP-NIr and saline-NIr groups would have had very similar cell numbers. Nevertheless, the reduction was clear and significant in the MPTP-NIr group and it may represent a reversal, at least in part, of the abnormal circuits generated by MPTP. It remains to be determined whether these changes in neuronal activity by NIr treatment are evident in behavioural and clinical studies.

In the MPTP and MPTP-NIr groups in both the STN and ZI, and saline-NIr group in the ZI, there were many Fos^+^ cells in the acute model with six-day survival and chronic model (three weeks survival). Previous studies have indicated that although the peak of Fos expression occurs about two hours after some forms of stimulation (e.g., peripheral noxious [17, 23]), it may still be present weeks or months after others (e.g., 6-OHDA lesion [25]). We show here that MPTP treatment had a long-term (six days to three weeks) impact on Fos expression, presumably due to the triggering and maintenance of abnormal basal ganglia circuitry (see above). Further, we show that even after minimal exposure, NIr treatment generated longer-term effects (six days to three weeks) on cell activity also. This finding is most encouraging when considering therapeutic use. In this context, it has been shown recently that patients suffering from depressive illness show improved depression and anxiety rating scales up to a month after a single dose of NIr treatment [20].

## Abbreviations

6-OHDA: 6-hydroxydopamine
ATP: Adenosine-5′-triphosphate
ic: Internal capsule
LG: Lateral geniculate nucleus
MG: Medial geniculate nucleus
MPTP: 1-methyl-4-phenyl-1,2,3,6-tetrahydropyridine
mt: Mammillothalamic tract
NIr: Near-infrared light treatment
PBS: Phosphate buffered saline
PpT: Pedunculopontine tegmental nucleus
Rt: Thalamic reticular nucleus
SCol: Superior colliculus
SNc: Substantia nigra pars compacta
SNr: Substantia nigra pars reticulate
STN: Subthalamic nucleus
TH: Tyrosine hydroxylase
ZI: Zona incerta
ZIc: Caudal sector zona incerta
ZId: Dorsal sector zona incerta
ZIr: Rostral sector zona incerta
ZIv: Ventral sector zona incerta.
